# Supplementary material for: COZOID: contact zone identifier for visual analysis of protein-protein interactions
Source: BMC Bioinformatics. 2018 Apr 6;19:125. doi: 10.1186/s12859-018-2113-6 (PMC5889581; doi:10.1186/s12859-018-2113-6)
Supplement: Supplementary file 2 — Software build. Executable binary file of the software tool. (ZIP 118,784 kb) [file 12859_2018_2113_MOESM2_ESM.zip › cozoid/platform/docs/testng-6.5.1-javadoc.zip!/org/testng/annotations/Test.html]

Test (TestNG)


---


|  |  |  |
| --- | --- | --- |
| **Overview** \| **Package** \| **Class** \| **Tree** \| **Deprecated** \| **Index** \| **Help** | |  |
| **PREV CLASS**   **NEXT CLASS** | **FRAMES**    **NO FRAMES**     **All Classes** |  |
| SUMMARY: REQUIRED \| OPTIONAL | DETAIL: ELEMENT |  |


---


## org.testng.annotations Annotation Type Test

---

``` @Retention(value=RUNTIME) @Target(value={METHOD,TYPE,CONSTRUCTOR}) public @interface Test ```

Mark a class or a method as part of the test.

**Author:**
:   Cedric Beust, Apr 26, 2004

---

| **Optional Element Summary** | |
| --- | --- |
| `boolean` | `alwaysRun`             If set to true, this test method will always be run even if it depends on a method that failed. |
| `java.lang.String` | `dataProvider`             The name of the data provider for this test method. |
| `java.lang.Class<?>` | `dataProviderClass`             The class where to look for the data provider. |
| `java.lang.String[]` | `dependsOnGroups`             The list of groups this method depends on. |
| `java.lang.String[]` | `dependsOnMethods`             The list of methods this method depends on. |
| `java.lang.String` | `description`             The description for this method. |
| `boolean` | `enabled`             Whether methods on this class/method are enabled. |
| `java.lang.Class[]` | `expectedExceptions`             The list of exceptions that a test method is expected to throw. |
| `java.lang.String` | `expectedExceptionsMessageRegExp`             If expectedExceptions was specified, its message must match the regular expression specified in this attribute. |
| `java.lang.String[]` | `groups`             The list of groups this class/method belongs to. |
| `boolean` | `ignoreMissingDependencies`             If set to true, this test will run even if the methods it depends on are missing or excluded. |
| `int` | `invocationCount`             The number of times this method should be invoked. |
| `long` | `invocationTimeOut`             The maximum number of milliseconds that the total number of invocations on this test method should take. |
| `java.lang.String[]` | `parameters`             **Deprecated.** *Use @Parameters* |
| `int` | `priority`             The scheduling priority. |
| `java.lang.Class` | `retryAnalyzer`             The name of the class that should be called to test if the test should be retried. |
| `boolean` | `sequential`             **Deprecated.** *Use singleThreaded* |
| `boolean` | `singleThreaded`             If set to true, all the methods on this test class are guaranteed to run sequentially, even if the tests are currently being run with parallel="true". |
| `boolean` | `skipFailedInvocations`             If true and invocationCount is specified with a value > 1, then all invocations after a failure will be marked as a SKIP instead of a FAIL. |
| `int` | `successPercentage`             The percentage of success expected from this method. |
| `java.lang.String` | `suiteName`             The name of the suite this test class should be placed in. |
| `java.lang.String` | `testName`             The name of the test this test class should be placed in. |
| `int` | `threadPoolSize`             The size of the thread pool for this method. |
| `long` | `timeOut`             The maximum number of milliseconds this test should take. |

### groups

```
public abstract java.lang.String[] groups
```

:   The list of groups this class/method belongs to.

    :   **Default:**: {}

---


### enabled

```
public abstract boolean enabled
```

:   Whether methods on this class/method are enabled.

    :   **Default:**: true

---


### parameters

```
@Deprecated
public abstract java.lang.String[] parameters
```

:   **Deprecated.** *Use @Parameters*

    :   The list of variables used to fill the parameters of this method.
        These variables must be defined in the property file.

        :   **Default:**: {}

---


### dependsOnGroups

```
public abstract java.lang.String[] dependsOnGroups
```

:   The list of groups this method depends on. Every method
    member of one of these groups is guaranteed to have been
    invoked before this method. Furthermore, if any of these
    methods was not a SUCCESS, this test method will not be
    run and will be flagged as a SKIP.

    :   **Default:**: {}

---


### dependsOnMethods

```
public abstract java.lang.String[] dependsOnMethods
```

:   The list of methods this method depends on. There is no guarantee
    on the order on which the methods depended upon will be run, but you
    are guaranteed that all these methods will be run before the test method
    that contains this annotation is run. Furthermore, if any of these
    methods was not a SUCCESS, this test method will not be
    run and will be flagged as a SKIP.
    If some of these methods have been overloaded, all the overloaded
    versions will be run.

    :   **Default:**: {}

---


### timeOut

```
public abstract long timeOut
```

:   The maximum number of milliseconds this test should take.
    If it hasn't returned after this time, it will be marked as a FAIL.

    :   **Default:**: 0L

---


### invocationTimeOut

```
public abstract long invocationTimeOut
```

:   The maximum number of milliseconds that the total number of invocations on this test
    method should take. This annotation will be ignored if the attribute invocationCount
    is not specified on this method.
    If it hasn't returned after this time, it will be marked as a FAIL.

    :   **Default:**: 0L

---


### invocationCount

```
public abstract int invocationCount
```

:   The number of times this method should be invoked.

    :   **Default:**: 1

---


### threadPoolSize

```
public abstract int threadPoolSize
```

:   The size of the thread pool for this method. The method will be invoked
    from multiple threads as specified by invocationCount.
    Note: this attribute is ignored if invocationCount is not specified

    :   **Default:**: 0

---


### successPercentage

```
public abstract int successPercentage
```

:   The percentage of success expected from this method.

    :   **Default:**: 100

---


### dataProvider

```
public abstract java.lang.String dataProvider
```

:   The name of the data provider for this test method.

    :   **See Also:**: `DataProvider`

        **Default:**: ""

---


### dataProviderClass

```
public abstract java.lang.Class<?> dataProviderClass
```

:   The class where to look for the data provider. If not
    specified, the dataprovider will be looked on the class
    of the current test method or one of its super classes.
    If this attribute is specified, the data provider method
    needs to be static on the specified class.

    :   **Default:**: java.lang.Object.class

---


### alwaysRun

```
public abstract boolean alwaysRun
```

:   If set to true, this test method will always be run even if it depends
    on a method that failed. This attribute will be ignored if this test
    doesn't depend on any method or group.

    :   **Default:**: false

---


### description

```
public abstract java.lang.String description
```

:   The description for this method. The string used will appear in the
    HTML report and also on standard output if verbose >= 2.

    :   **Default:**: ""

---


### expectedExceptions

```
public abstract java.lang.Class[] expectedExceptions
```

:   The list of exceptions that a test method is expected to throw. If no
    exception or a different than one on this list is thrown, this test will be
    marked a failure.

    :   **Default:**: {}

---


### expectedExceptionsMessageRegExp

```
public abstract java.lang.String expectedExceptionsMessageRegExp
```

:   If expectedExceptions was specified, its message must match the regular expression
    specified in this attribute.

    :   **Default:**: ".\*"

---


### suiteName

```
public abstract java.lang.String suiteName
```

:   The name of the suite this test class should be placed in. This
    attribute is ignore if @Test is not at the class level.

    :   **Default:**: ""

---


### testName

```
public abstract java.lang.String testName
```

:   The name of the test this test class should be placed in. This
    attribute is ignore if @Test is not at the class level.

    :   **Default:**: ""

---


### sequential

```
public abstract boolean sequential
```

:   **Deprecated.** *Use singleThreaded*

    :   **Default:**: false

---


### singleThreaded

```
public abstract boolean singleThreaded
```

:   If set to true, all the methods on this test class are guaranteed to run
    sequentially, even if the tests are currently being run with parallel="true".
    This attribute can only be used at the class level and will be ignored
    if used at the method level.

    :   **Default:**: false

---


### retryAnalyzer

```
public abstract java.lang.Class retryAnalyzer
```

:   The name of the class that should be called to test if the test
    should be retried.

    :   **Returns:**: String The name of the class that will test if a test method should be retried.

        **Default:**: java.lang.Class.class

---


### skipFailedInvocations

```
public abstract boolean skipFailedInvocations
```

:   If true and invocationCount is specified with a value > 1,
    then all invocations after a failure will be marked as a SKIP
    instead of a FAIL.

    :   **Default:**: false

---


### ignoreMissingDependencies

```
public abstract boolean ignoreMissingDependencies
```

:   If set to true, this test will run even if the methods
    it depends on are missing or excluded.

    :   **Default:**: false

---


### priority

```
public abstract int priority
```

:   The scheduling priority. Lower priorities will be scheduled first.

    :   **Default:**: 0


---


|  |  |  |
| --- | --- | --- |
| **Overview** \| **Package** \| **Class** \| **Tree** \| **Deprecated** \| **Index** \| **Help** | |  |
| **PREV CLASS**   **NEXT CLASS** | **FRAMES**    **NO FRAMES**     **All Classes** |  |
| SUMMARY: REQUIRED \| OPTIONAL | DETAIL: ELEMENT |  |


---
